# A rare case of jejunal Vanek’s tumor causing intussusception in an adult: a case report and comprehensive literature overview

**DOI:** 10.1093/jscr/rjad642

**Published:** 2023-12-05

**Authors:** Nicolò Fabbri, Francesco Rimi, Valentina Sani, Antonio Pesce, Salvatore Greco, Stefano Gobbo, Carlo V Feo

**Affiliations:** Unit of General Surgery, Local Health Agency of Ferrara, Ferrara, Emilia-Romagna, Italy; Department of Medical Science, University of Ferrara, Ferrara, Italy; Department of Medical Science, University of Ferrara, Ferrara, Italy; Unit of General Surgery, Local Health Agency of Ferrara, Ferrara, Emilia-Romagna, Italy; Department of Translational Medicine, University of Ferrara, Ferrara, Italy; Department of Translational Medicine, University of Ferrara, Ferrara, Italy; Unit of General Surgery, Local Health Agency of Ferrara, Ferrara, Emilia-Romagna, Italy; Department of Medical Science, University of Ferrara, Ferrara, Italy

**Keywords:** inflammatory fibroid polyp, intussusception, Vanek’s tumor, case report

## Abstract

Inflammatory fibroid polyp, or Vanek's tumor, is an uncommon benign small bowel tumor and a rare cause of intussusception in adults. This case involves a 62-year-old man with persistent abdominal pain, diagnosed with jejunoileal intussusception. A 4 cm inflammatory fibroid polyp was discovered during surgery, leading to distal jejunal resection. Despite the rarity of adult intussusceptions, they should be considered in abdominal pain diagnoses. The optimal management approach, whether *en bloc* resection or initial reduction with limited resection, remains debated.

## Introduction

Inflammatory fibroid polyp (IFP) is an unusual, idiopathic, benign gastrointestinal tumor, classified as a submucosal connective tissue tumor. Vanek [1] first described this tumor in the stomach and it can affect both sexes (with a slight predominance in males) of any age, with higher incidence in the fifth and seventh decades [2, 3]. IFPs can be found most commonly in the gastric antrum (0.1%–3.0% of all gastric polyps) [3] or ileum, rarely in the duodenum and jejunum without a certain cause.

The suggested etiologies are related to chemical, physical, or metabolic triggers [4] even if in some cases, a genetic study of IFP allowed to detect mutations in platelet-derived growth factor alpha. Most IFPs have diameters ranging from 3 to 4 cm at the time of diagnosis and frequently are solitary polyps [5].

Rarely, IFP could coexist in a Crohn disease (6). The symptoms include abdominal pain with or without vomit, altered small bowel peristalsis, gastrointestinal bleeding, and/or weight loss depending on both localization and size of the lesion.

IFPs arising below the Treitz ligament can cause acute abdomen for obstructive ileus, usually because of intussusception [6, 7], which can rapidly evolve into a surgical emergency. The preoperative diagnosis of intussusception is rare but can be reached through imaging techniques [8] such as ultrasound scanning, even if abdominal computed tomography (CT) is currently considered the gold standard technique for detecting the polyp or for confirming the intussusception [5].

Small bowel intussusception in adults has to be always considered a pathological condition, and surgical intervention is strongly recommended [9]. Furthermore, exploratory laparoscopy or laparotomy is frequently recommended as the best treatments for intussusception, in order to prevent bowel ischemia, necrosis, and subsequent perforation of the invaginated bowel segment [5]. Immunohistochemistry is used to distinguish between IFPs (expressing proteins on their cellular surface such as CD34 and Vimentin) and other benign tumors, such as gastrointestinal stromal tumors (GIST), positive for CD34 and Vimentin but also for CD117.

We present the following case, in accordance with the CARE reporting checklist, concerning an adult man operated for intussusception with the finding of a rare jejunal Vanek’s tumor with a literature overview.

## Methods

We performed a double literature research (without year filters nor patient’s age) using the PubMed database with the following codes:

- (inflammatory fibroid polyp) AND (small bowel): 156 results;
- (Vanek) AND (small bowel): 31 results.

Of them, 23 articles were removed because of duplicates. Subsequently, we excluded all articles regarding “ileal” or “ileo-ciecal” IFPs with or without intussusception, focusing on the jejunal tumors. Only 15 articles were found (Table 1).

**Table 1:** Jejunal Vanek’s cases. A PubMed literature review.

**Author, year, journal**	**DOI/PMID**	**Type of study**	**Age**	**Sex**	**Symptoms**	**Findings**	**Type of surgery**
Kim JS, *et al.1994* Korean J Intern Med	10.3904/kjim.1994.9.1.51	case report	52	female	intermittent abdominal pain, vomiting	Intussusception diagnosed via CT	laparotomy
Ling CC, *et al.* 1994 Zhonghua Yi Xue Za Zhi (Taipei)	8167990	case report	56	female	intermittent abdominal pain, vomiting, diarrhea	Intussusception diagnosed via CT	laparotomy
Shih LN, *et al.* 1997 Am J Gastroenterol.	8995961	case report	66	male	abdominal fullness, pain	Intussusception diagnosed via CT	laparotomy
Kuestermann SA *et al.* 1999 Radiographics	10.1148/radiographics.19.2.g99mr19539	case report	34	female	intermittent abdominal pain, vomiting	Intussusception diagnosed via CT	laparotomy
Sah SP, *et al.* 2002 Indian J Pathol Microbiol.	12593579	case report	45	male	abdominal fullness, vomiting	Intussusception diagnosed via CT	laparotomy
Topaloglu S *et al.* 2003 Hepatogastroenterology	15244194	case report	56	male	Not found	Intussusception diagnosed via CT	Not found
Cipe G, *et al.*2009 Cases J.	10.4076/1757-1626-2-6435	case report	31	male	nausea and vomiting	Intussusception diagnosed via CT	laparotomy
Rehman S, *et al.* 2009 Cases J.	10.1186/1757-1626-2-7152	case report	46	male	abdominal pain	Intestinal obstruction	laparotomy
Neishaboori H, *et al.* 2013 Gastroenterol Hepatol Bed Bench.	24834274	case report	40	female	abdominal pain, vomiting	Intussusception diagnosed via CT	laparotomy
Joyce KM, *et al.* 2014 Diagn Pathol.	10.1186/1746-1596-9-127	case report	62	male	pseudo-obstruction, small bowel obstruction two weeks later	Intussusception, no tumor detected	Laparoscopic reduction, subsequently, laparotomic resection
Kang SH, *et al.* 2015 Ann Coloproctol.	10.3393/ac.2015.31.3.106	case report	51	female	abdominal pain	Intussusception diagnosed via CT	laparotomy
Kameda C, *et al.* 2018 Clin Endosc	10.5946/ce.2017.162	case report	68	female	Anemia, positive fecal occult blood test	Tumor diagnosed via Capsule Endoscopy	Laparoscopic resection
Park KB, *et al.* 2020 Int J Surg Case Rep.	10.1016/j.ijscr.2020.03.029	case report	23	male	epigastric pain	Intussusception diagnosed via CT	Laparoscopic resection
Sverrisdottir V, *et al.* 2020 Laeknabladid	10.17992/lbl.2020.0708.591	case report	25	female	abdominal pain, anemia	Tumor diagnosed via duodenoscopy	Converted laparoscopy
Tan JS, *et al.* 2020 Ann Med Surg (Lond)	10.1016/j.amsu.2020.10.009	case report	20	female	abdominal pain	Intussusception with bowel ischemia	laparotomy
Fabbri N. *et al.* 2023	—	review	62	male	abdominal pain	Intussusception diagnosed via CT	laparotomy

Jejunal Vanek's cases: a PubMed literature review. Age and sex of patients, clinical presentation and surgical intervention.

## Case presentation

A 62-years-old man with a medical history of diabetes mellitus, hypertension, chronic gastritis, and cholelithiasis was admitted to the emergency room (ER) for persistent cramping abdominal pain, began about 40 days earlier. He was first discharged at home after the visit and an abdominal X-ray (showing rare hydro-aerial levels only) with a diagnosis of “undetermined acute colitis.”

The patient decided to get back to ER for the exacerbation of the symptoms and diaphoresis, without manifesting nausea, vomit, or bowel movement pattern alterations. The physical examination of the abdomen revealed abdominal guard and pain localized in the lower quadrants of the abdomen.

He was later admitted to the Short-Stay Emergency Department Observation Unit, where a second abdominal X-ray was performed, with the finding of fluid levels in the upper left quadrant.

**Figure 1:**
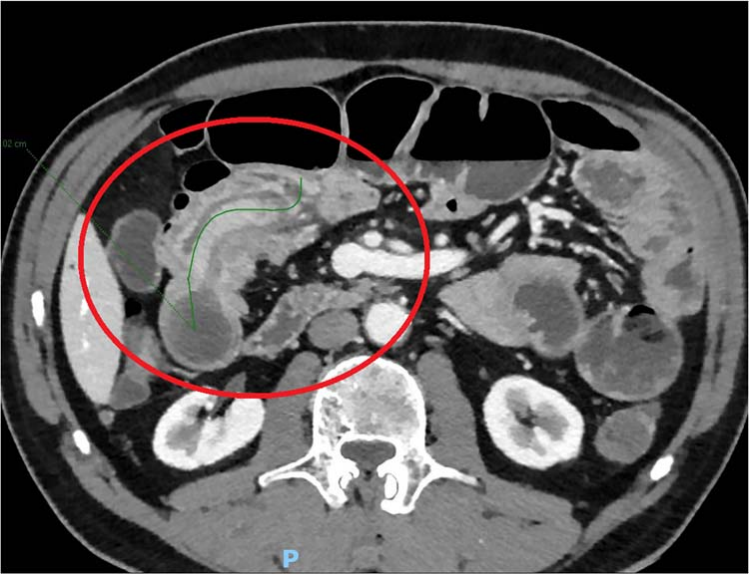
Abdominal TC with the details of intussusception.

The day after, the new abdominal X-ray documented increasing distension of the bowel, with multiple hydro-aerial levels and it was decided to perform an abdominal CT scan with iodinated contrast agent: this revealed an ileo-ileal invagination extending in the mesogastrium region for about 10 cm, without showing the characteristics of expansive neoformations (Fig. 1).

The laboratory exams showed only mild neutrophilic leukocytosis, without increased inflammatory indexes. The patient was finally admitted to the surgical department for urgent laparotomy and the intraoperative findings revealed an intussusception involving a stretch of about 10 cm and supported by a 4 cm thick, rounded tense-elastic neoformation, possibly originated from the intestinal wall. A segmental jejunal resection and side-to-side mechanical anastomosis were performed.

The clinical course was complicated by an episode of enterorrhagia that required a blood transfusion of 1 unit of concentrated red cells, but the patient was discharged after 11 days in good clinical conditions, without manifesting abdominal symptoms, even after 6 months of telephonic follow-up.

**Figure 2:**
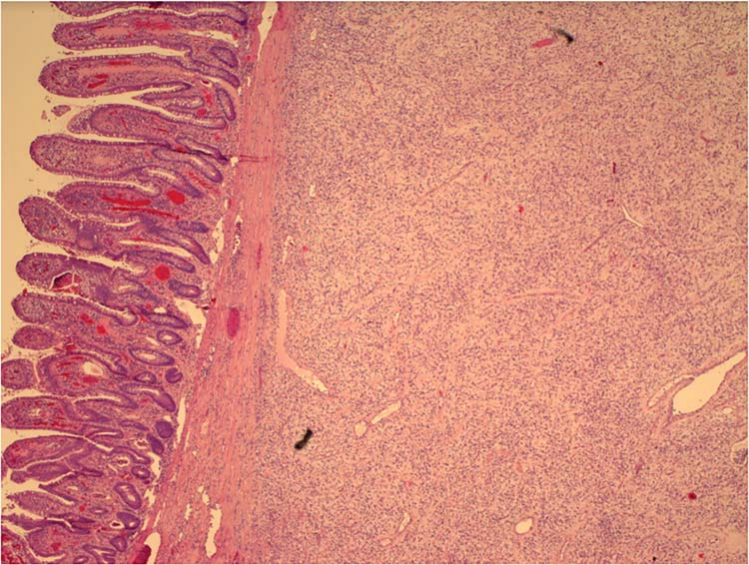
Histological image of Vanek’s tumor with intact intestinal mucosa.

Histological examination later showed a 3.5 cm grayish color mass without aspects of cellular atypia or mitotic activity; the immunohistochemical analysis did not present aspects referable to GIST or leiomyomatosis; the overall morphological and immunophenotypic picture allowed the diagnosis of IFP (Vanek’s tumor) (Fig. 2). “All procedures performed in this study were in accordance with the ethical standards of the institutional and/or national research committee(s) and with the Helsinki Declaration (as revised in 2013).”

## Discussion

IFPs were first described in the stomach by the Czech pathologist Josef Vanek, in 1949: the first IFP was an indolent, non-capsulated, submucosal granuloma, composed mainly of loose connective tissues, vessels, and with an eosinophilic inflammatory component [5]. The name “inflammatory fibroid polyp” was later introduced by Helvig and Renier [10] to indicate the nonneoplastic nature of this tumor, even if the theoretical neoplastic nature of IFP was hypothesized by Schildhaus *et al.* [11], who discovered a relation between this tumor and mutations of platelet-derived growth factor receptor A proto-oncogene. IFPs are infrequent, idiopathic, benign, neoplasms primarily originating in the gastrointestinal submucosa [5], whereas the involvement of small intestine and colon by IFPs is rare [1–10].

The most common site of development by IFPs is the gastric antrum (66%–75%), followed by small intestine (18%–20%), colorectal region (4%–7%), gallbladder (1%), esophagus (1%), duodenum (1%), and appendix (<1%). The ileal tract, however, is the site where such tumors cause more often intussusception [12, 13]. While the exact pathogenesis remains unknown, some triggers as trauma, allergic reactions, genetic predisposition, infections, physical, chemical, or metabolic stimuli could be initiators of the process. For example, a traumatic etiology is possible for gastric lesions, because of the coarse food content and muscular contractions, but it is difficult to explain the lesions in the small bowel on this basis. Macroscopically, the mucosal surface is usually ulcerated and pale; microscopically, the tumor is composed of mononuclear, spindle-shaped cells, which are characteristically arranged in whorls or in an onion-skin-like fashion around blood vessels or mucosal glands, forming a “whirl-like” structure. The “classic” (or gastric) type originally described by Vanek is characterized by heavy inflammatory infiltration, rich in eosinophilic granulocytes. There is no considerable proliferative activity as mitosis of the spindle cell is rarely detected and the Ki-67 index is usually lower than 1% (indicating, thus, a low probability of malignant degeneration). Immunostaining for other specific markers such as KIT, DOG-1, S-100, and EMA is generally consistently negative and this may be important in the differential diagnosis of GIST, peri-neurinoma, and other spindle cell lesions of the gastrointestinal tract. IFPs are usually asymptomatic and could remain undiagnosed for long time or can be incidentally found during endoscopic procedures or laparotomy. When such polyps become symptomatic, the clinical symptoms depend on both localization and size of the tumor [14]. Abdominal pain is the main symptom in patients with lesions in the stomach [15], whereas patients with IFPs in the small bowel are most likely to present with chronic episodes of colicky abdominal pain, lower gastroenteric bleeding with or without anemia, and, more rarely, intestinal obstruction because of episodes of intestinal intussusception [5], as happened in this case. An accurate diagnosis is based on physical examination and specific imaging: this includes X-rays, ultrasonography (US), CT scans, magnetic resonance imaging, enteroclysis, endoscopic procedures, angiography, and use of video capsule [11–16]. Abdominal CT is currently considered the most sensitive radiological method for polyp detection, and can be also able to confirm intussusception, with reported diagnostic accuracy of 58%–100%; differently from US, CT is, in fact, unaffected by the presence of gas in the bowel lumen [5]. The etiology of intussusceptions in small bowel and colon is quite different: in the small intestine there is a predominance of benign processes, with up to 90% of cases including hamartomas, lipomas, leiomyomas neurofibromas, adenomas, Peutz–Jeghers syndrome, adhesions, and only rarely IFPs [14]. Malignant lesions (sarcomas, lymphomas, and carcinoid tumors or metastatic deposits from melanoma, breast, and lung cancers) account for 14%–47% of all cases of intussusception in the small intestine. The treatment of most IFPs is generally performed in course of endoscopy with submucosal dissection [17], whereas open or laparoscopic surgery is rarely needed. While on one hand it is true that surgical reduction before resection may theoretically allow more limited resections, the risk of potential intraluminal seeding or venous tumoral dissemination during the manipulation of a malignant lesion should also be taken into consideration. The final decision concerning eventual surgery should be necessarily taken based on the nature of such lesions and of the symptoms associated. When a preoperative diagnosis of a benign lesion is safely established, the surgeon may reduce the intussusception by milking it out in a distal-to-proximal direction: this may be carried out by using laparoscopy in suitable cases. Reduction should not be attempted if there are signs of inflammation or ischemia of the intestinal wall. The optimal surgical management of adults with small bowel intussusception varies between reduction and resection: reduction can be attempted, in small bowel intussusception, only when the segment involved is certainly viable and malignancy is not suspected [18].

Based on our literature overview (Table 1), jejunal Vanek’s tumor occurs equally in both sex and often presents with a mean age of 46 years (range 20–68) and persistent abdominal pain for several days. CT rarely reveals the tumor, while intussusception is often present. Jejunal segmental laparotomic resection remains the first choice of treatment.

## Conclusion

Intussusceptions, although rare in adults (1%–5% of all cases of intestinal obstruction), have to be considered in the differential diagnosis of abdominal pain.

Exploratory laparoscopy or laparotomy is frequently recommended as the best treatment for intussusceptions caused by IFPs; surgery should be performed as early as possible in order to prevent the intussusception’s complications such as ischemia, necrosis, and perforation of the invaginated bowel segment. When the operation is delayed and intestinal perforation with peritonitis occurs, there is a considerable increase in morbidity and mortality.

The appropriate management of intussusceptions in adults remains controversial, and the debate focuses mostly on the choice between primary *en bloc* resection and initial reduction followed by more limited resection. Thus, the most important factors in the surgical decision process before histologic diagnosis of the lesions are localization and size of the mass and viability of the invaginated segment [14, 19, 20].

Much more evidence concerning Vanek’s tumors is necessary to improve our knowledge on such particularly rare lesions.

## Author contributions

N.F., F.R., V.S., A.P., and S.G. obtained patient’s consent, reviewed literature, and drafted the manuscript. C.V.F. and N.F. were the surgeons on the case. They reviewed the manuscript, as senior authors. S.G. was the pathologist on the case. He reviewed and edited the manuscript.

## Conflict of interest statement

Authors confirm this is original research that has not been published elsewhere. Authors have no relevant financial or nonfinancial conflict of interest to disclose.

## Funding

No funds, grants, or other support were received for this study.

## Data availability

This patient’s non-nominal data are available from corresponding author (N.F.), upon reasonable request.

## Informed consent

The patient here in presented gave informed written consent for this case report to be shared and published.

## Ethics approval

For this case report, formal approval by the Research Ethics Committee of area Vasta di Bologna (Avec-CE) was waived.

## References

[1] Vanek J . Gastric submucosal granuloma with eosinophilic infiltration. Am J Pathol 1949;25:397–411. 18127133 PMC1942901

[2] Liu TC, Lin MT, Montgomery EA, Singhi AD. Inflammatory fibroid polyps of the gastrointestinal tract: spectrum of clinical, morphologic, and immunohistochemistry features. Am J Surg Pathol 2013;37:586–92. 23426127 10.1097/PAS.0b013e31827ae11e

[3] Carmack SW, Genta RM, Schuler CM, Saboorian MH. The current spectrum of gastric polyps: a 1-year national study of over 120,000 patients. Am J Gastroenterol 2009;104:1524–32. 19491866 10.1038/ajg.2009.139

[4] Kolodziejczyk P, Yao T, Tsuneyoshi M. Inflammatory fibroid polyp of the stomach. A special reference to an immunohistochemical profile of 42 cases. Am J Surg Pathol 1993;17:1159–68. 8214261 10.1097/00000478-199311000-00009

[5] Abboud B . Vanek's tumor of the small bowel in adults. World J Gastroenterol 2015;21:4802–8. 25944993 10.3748/wjg.v21.i16.4802 PMC4408452

[6] Parasi A, Triantafillidis JK, Barbatzas C, Karakosta A, Condilis N, Sotiriou H. Coexistence of Crohn’s disease and inflammatory fibroid polyp of the small bowel. Report of a case and review of the literature. Ann Ital Chir 2005;76:395–9. 16550878

[7] Zager JS, Shaw JP, Kaufman JP, DeNoto G. Three cases of small bowel intussusception in relation to a rare lesion: inflammatory fibrous polyps. Dig Surg 2001;18:142–6. 11351161 10.1159/000050116

[8] Jabar MF, Prasannan S, Gul YA. Adult intussusception secondary to inflammatory polyps. Asian J Surg 2005;28:58–61. 15691802 10.1016/S1015-9584(09)60262-1

[9] Jacob S, Lee T, Yuen L. Rare case of small bowel intussusception secondary to an inflammatory fibroid polyp (Vanek's tumour). ANZ J Surg 2021;91:E673–4. 33634595 10.1111/ans.16709

[10] Johnstone JM, Morson BC. Inflammatory fibroid polyp of the gastrointestinal tract. Histopathology 1978;2:349–61. 721077 10.1111/j.1365-2559.1978.tb01727.x

[11] Schildhaus HU, Cavlar T, Binot E, Büttner R, Wardelmann E, Merkelbach-Bruse S. Inflammatory fibroid polyps harbour mutations in the platelet-derived growth factor receptor alpha (PDGFRA) gene. J Pathol 2008;216:176–82. 18686281 10.1002/path.2393

[12] Helwig EB, Ranier A. Inflammatory fibroid polyps of the stomach. Surg Gynecol Obstet 1953;96:335–67. 13038651

[13] Bays D, Anagnostopoulos GK, Katsaounos E, Filis P, Missas S. Inflammatory fibroid polyp of the small intestine causing intussusception: a report of two cases. Dig Dis Sci 2004;49:1677–80. 15573926 10.1023/b:ddas.0000043385.44842.c8

[14] Akbulut S . Intussusception due to inflammatory fibroid polyp: a case report and comprehensive literature review. World J Gastroenterol 2012;18:5745–52. 23155316 10.3748/wjg.v18.i40.5745 PMC3484344

[15] Nonose R, Valenciano JS, da Silva CM, de Souza CAF, Martinez CAR. Ileal Intussusception Caused by Vanek’s Tumor: A Case Report. Case Rep Gastroenterol 2011;5:110–6. 21503167 10.1159/000326930 PMC3078240

[16] Beall DP, Fortman BJ, Lawler BC, Regan F. Imaging bowel obstruction: a comparison between fast magnetic resonance imaging and helical computed tomography. Clin Radiol 2002;57:719–24. 12169282 10.1053/crad.2001.0735

[17] Shaib YH, Rugge M, Graham DY, Genta RM. Management of gastric polyps: an endoscopy-based approach. Clin Gastroenterol Hepatol 2013;11:1374–84. 23583466 10.1016/j.cgh.2013.03.019 PMC3962745

[18] Joyce KM, Waters PS, Waldron RM, Khan I, Orosz ZS, Németh T, et al. Recurrent adult jejuno-jejunal intussusception due to inflammatory fibroid polyp - Vanek's tumour: a case report. Diagn Pathol 2014;9:127. 24968941 10.1186/1746-1596-9-127 PMC4094443

[19] Wang N, Cui XY, Liu Y, Long J, Xu YH, Guo RX, et al. Adult intussusception: a retrospective review of 41 cases. World J Gastroenterol 2009;15:3303–8. 19598308 10.3748/wjg.15.3303 PMC2710788

[20] Marinis A, Yiallourou A, Samanides L, Dafnios N, Anastasopoulos G, Vassiliou I, et al. Intussusception of the bowel in adults: a review. World J Gastroenterol 2009;15:407–11. 19152443 10.3748/wjg.15.407 PMC2653360

